# Can aromatherapy reduce restless legs syndrome in hemodialysis patients? a systematic review and meta-analysis

**DOI:** 10.7586/jkbns.24.022

**Published:** 2024-08-27

**Authors:** Mi-Kyoung Cho, Mi Young Kim

**Affiliations:** 1 Department of Nursing Science, Research Institute of Nursing Science, Chungbuk National University, Cheongju, Korea; 2 College of Nursing, Hanyang University, Seoul, Korea

**Keywords:** Restless legs syndrome, Renal dialysis, Aromatherapy, Meta-analysis, Systematic review

## Abstract

**Purpose:**

This study conducted a systematic literature review and meta-analysis on the effectiveness of aromatherapy in reducing restless legs syndrome (RLS) in hemodialysis patients.

**Methods:**

Using the population, intervention, comparison, outcome, study design framework, a search was conducted of eight electronic databases: PubMed, Cochrane, Embase-Ovid, CINAHL, Web of science, Scopus, PQDT, and RISS. The population was hemodialysis patients, and the intervention included aromatherapy aiming at RLS, compared to control groups receiving placebo or usual care. The outcome measured was RLS, and the study design was randomized controlled trials.

**Results:**

The analysis included seven articles presenting results from 10 studies, and the pooled overall effect of aromatherapy on RLS in hemodialysis patients was shown by a Hedge’s g of −1.84 (95% confidence interval: −2.45 to −1.23). Meta-regression analysis revealed greater effectiveness in studies that received funding. Age over 60, lavender oil use, intervention duration of less than 4 weeks, sessions longer than 30 minutes, a routine care control group, and quality assessment score of 10 or less were associated with RLS reduction.

**Conclusion:**

The study concluded that aromatherapy is effective for managing RLS in hemodialysis patients.

## INTRODUCTION

One of the essential treatments for kidney disease patients to survive is hemodialysis, which is the most widely used treatment among patients. However, there can be various side effects associated with hemodialysis. A significant one is sleep disturbance, with 85%-99% of patients reporting sleep disorders according to previous studies [1]. Most hemodialysis patients experience decreased quality of sleep, leading to an increased risk of accidents, falls, fatigue, and functional decline, with restless legs syndrome (RLS) also occurring in these patients [2]. Potential causes of muscle cramps in hemodialysis patients include high ultrafiltration, hypotension, hyponatremia, electrolyte imbalance, and impaired muscle energy metabolism due to carnitine deficiency, which is strongly associated with RLS [3].

RLS is a neurological disorder characterized by an irresistible urge to move the legs, accompanied by unpleasant sensations such as tingling, burning, crawling, or aching feelings [4]. The prevalence of undiagnosed RLS in hemodialysis patients is reported to be 28%-62% [5]. RLS is a very uncomfortable sensation that includes pain, tingling, itching, burning, numbness, tingling, or a feeling of discomfort that is not particularly painful [6]. RLS has also been associated with reduced quality of life, decreased work productivity, and significant social and economic burdens [7].

As the impact of RLS is negative, intervention for RLS is essential. While pharmacological treatments are often chosen for severe RLS cases, they can sometimes cause side effects [8]. Therefore, the usefulness of non-pharmacological treatments is increasingly being reported, including exercise, reflexology, massage, and complementary and integrative therapies such as aromatherapy [9]. Aromatherapy, which utilizes essential oils extracted from aromatic herbs, has a long history and is known to impact on physical and mental health [10]. One of the essential oils used in aromatherapy is reported to have anti-anxiety, anti-depressant, calming, muscle fatigue reduction, blood circulation, and quality of life improvement effects [11,12]. There are also reports of its effectiveness in reducing stress and spastic movements [13]. Aromatherapy is known for its easy absorption through the skin via aromatic oils and plant volatile substances. It has been reported to have calming and muscle relaxation effects without side effects [14]. It may be effective in managing spasticity and RLS in hemodialysis patients.

Therefore, a comprehensive review is needed to determine whether aromatherapy can be effective for RLS, one of the side effects experienced by dialysis patients. Additionally, specific application methods of aromatherapy, such as the type of oil, application method, and duration, need to be systematically reviewed and analyzed to determine the most effective approach. In this study, we aimed to conduct a systematic review and meta-analysis to investigate the effects of aromatherapy on reducing RLS in hemodialysis patients, in order to provide fundamental data on complementary and alternative therapies.

## METHODS

### Search strategy and data sources

The literature search was conducted by two researchers, Cho, M.-K. and Kim, M.Y., from the inception of the papers to June 30, 2024. The search was performed in 8 electronic databases: PubMed, Cochrane, Embase-Ovid (Excerpta Medica Database), CINAHL (The Cumulative Index to Nursing and Allied Health Literature), WoS (Web of science), Scopus, PQDT (The ProQuest Dissertations & Theses Global), and RISS (Research Information Service System). The search included articles published in English and Korean. The literature search was conducted from June 29, 2024, to July 8, 2024 (Appendix 1). The search strategy and protocol were based on the PICO-SD (population, intervention, comparison, outcome-study design) framework and are available at CRD42024559837 (https://www.crd.york.ac.uk/prospero/display_record.php?RecordID=559837).

### Inclusion and exclusion criteria

The selection criteria for this study were as follows: The study population (P) was hemodialysis patients aged 19 years and older, the intervention (I) was aromatherapy, the control (C) was conventional care or a comparative intervention, and the outcome (O) was RLS (Appendix 1). In cases where a single paper reported on two or more interventions (study ID: 1, 3, 7), the effect size for each intervention was calculated separately. The selection criteria included studies reported in either Korean or English with available full-text, and studies that provided the necessary data (sample size, mean, and standard deviation) to calculate the pooled effect size. The SD included randomized controlled trials (RCTs) and a quasi-experimental study. The exclusion criteria included: studies where subjects were participants under 19 years of age; studies where RLS was not reported as an outcome; studies where the experimental intervention was not aromatherapy; unpublished manuscripts; conference abstracts; protocols; and single-group studies without a control group.

### Data extraction

According to the inclusion and exclusion criteria, two researchers independently searched the data to select studies for analysis. The selected studies were recorded in a coding book created using Microsoft Excel spreadsheet software. The extracted information included: author, publication year, country, Institutional Review Board (IRB), funding source, number of subjects, SD, intervention characteristics (type of oil, properties and amount of oil used, application site and method, interventionist, intervention duration, number of sessions, time per session, control group intervention, timing of post-test measurement), dependent variables, and measurement tools. In case of coding discrepancies, the original articles were reviewed again, and final coding values were determined (Table 1, Appendix 2).

### Quality assessment

The selected studies were all of RCT design, and the quality assessment of the selected literature was independently conducted by two researchers. The seven articles included in the analysis had an average quality score based on the 13-item JBI Checklist for RCTs tool [15]. For the items 'Q2. Was allocation to treatment groups concealed?' (study ID: 1), 'Q4. Were participants blind to treatment assignment?', and 'Q5. Were those delivering the treatment blind to treatment assignment?' (study ID: 4), only one article reported. For the item 'Q7. Were outcome assessors blind to treatment assignment?', only four studies (study ID: 1, 2, 4, 5) were clearly reported. All other quality assessment items were reported in all the studies (Appendix 3).

### Statistical analysis

MIX 2.0 Pro (Ver. 2.0.1.6, BiostatXL, 2017) was used to calculate and merge effect sizes for RLS, the primary outcome of the studies. For the pooled overall effect of RLS, Hedge's g was used as the effect size due to the small number of studies, and a synthesis forest plot was used using a random effects model with reweighting to account for variations in subject characteristics and study-specific heterogeneity. The significance of the effects was determined using the 95% confidence intervals (CI) and a p-value less than .05. The weights of the individual effect sizes were derived using the inverse [16]. The heterogeneity of the included studies was assessed using the Q statistics and Higgin's I^2^ values [17]. Considering the potential bias in the point estimate of I^2^ in small meta-analyses, the 95% CI for I^2^ was also reported. Heterogeneity was interpreted as present when I^2^ exceeded 50% [18]. To identify the factors determining the heterogeneity in the studies on the effect of aromatherapy on RLS in hemodialysis patients, subgroup analysis, meta-regression, and exclusion sensitivity analysis were performed [19]. Publication bias was also tested using funnel plots, trim and fill plots, and the trim and fill method, and the pooled overall effect was adjusted accordingly [20].

## RESULTS

### Selection and characteristics of the included studies

**Figure 1.**
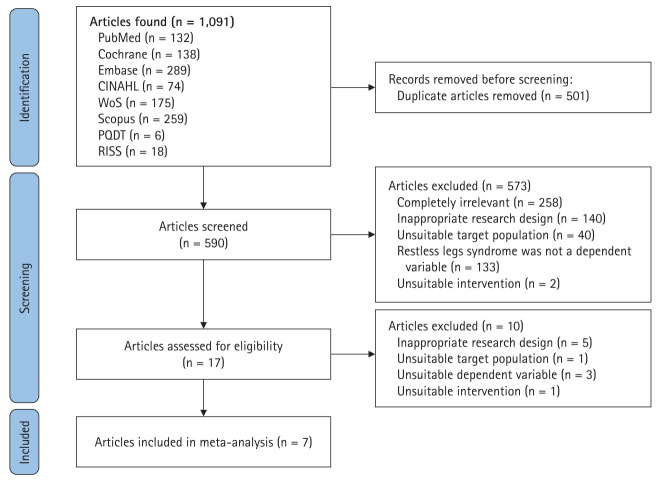
PRISMA flow diagram.

In this study, the selection of the analysis target studies was conducted in a three-step process following the preferred reporting items for systematic review and meta-analysis guidelines, as illustrated in Figure 1. In the first stage of identification, a total of 1,091 studies were searched through 8 databases based on the search strategy. In the second stage of screening, 590 studies were extracted after excluding duplicates, and 17 papers were selected according to the inclusion and exclusion criteria. In the third stage of inclusion, a total of 10 studies were selected as the final analysis targets, including seven research papers and two studies from one paper (study ID: 1, 3, 7).

The characteristics of the analysis target papers are as follows: four studies were published in 2021 or later (study ID: 1, 2, 6, 7), six studies were conducted in Iran (study ID: 1, 2, 3, 4, 5, 7), three studies were conducted in more than two centers (study ID: 3, 5, 6), one study received IRB approval (study ID: 2) and one study did not receive funding (study ID: 6), four studies had 60 or more participants (study ID: 1, 3, 4, 7), and all seven studies were RCTs (Table 1).

The characteristics of the interventions in the analysis target studies are as follows: all studies applied aromatherapy through massage, with the application site being the leg in five studies (study ID: 3^a^, 3^b^, 4, 5, 6) and the feet in five studies (study ID: 1^a^, 1^b^, 2, 7^a^, 7^b^), and lavender oil being used in six studies (study ID: 1^a^, 2, 3^a^, 5, 6, 7^a^). seven studies used more than 10 mL of oil (study ID: 2, 3^a^, 3^b^, 4, 5, 7^a^, 7^b^), three studies had the aromatherapy massage applied by an aromatherapist (study ID: 1^a^, 1^b^, 6), six studies had an intervention duration of 4 weeks or more and 9 or more intervention sessions (study ID: 1^a^, 1b, 2, 3^a^, 3^b^, 6), and seven studies had an intervention time of less than 30 minutes per session (study ID: 1^a^, 1^b^, 2, 3^a^, 3^b^, 7^a^, 7^b^). Six studies had a control group that received an intervention other than usual care (study ID: 1^a^, 1^b^, 3^a^, 3^b^, 4, 6), five studies measured the dependent variables immediately after the intervention (study ID: 1^a^, 1^b^, 6, 7^a^), and five studies had a quality assessment score of 10 or higher (study ID: 1^a^, 1^b^, 2, 4, 5) (Table 1, 2).

### The effect of aromatherapy on RLS among hemodialysis patients

**Figure 2.**
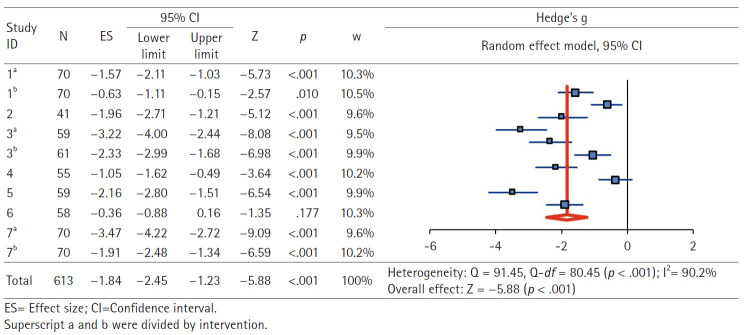
The efffect of aromatherapy on restless syndrom among hemodialysis patients.

The pooled overall effect of aromatherapy on RLS in hemodialysis patients was Hedge's g = −1.84 (95% CI: −2.45 to −1.23), indicating a large effect according to the interpretation criteria proposed by Brydges [21] (Figure 2). The heterogeneity test showed Q = 91.45 (Q-*df* = 80.45, *p* < .001), and Higgins' I^2^ was 90.2%, suggesting a high degree of heterogeneity among the studies. Consequently, subgroup analysis and univariate meta-regression were conducted to explore the sources of heterogeneity.

In the subgroup analysis, the study characteristics were categorized by funding, number of participants (cutoff at 60), intervention method, application area of aroma massage (feet vs. leg), oil type (lavender vs. other oils), oil amount (cutoff at 10 mL), oil products (commercial vs. handmade), control group intervention (usual care vs. placebo), facilitator (nurse/researcher vs. aromatherapist), intervention duration (cutoff at 4 weeks), intervention sessions (cutoff at 9 sessions), time per session (cutoff at 30 minutes), measurement time of post-test (immediate vs. delayed), and quality score (cutoff at 10 points). The pooled effect size, 95% CI, and significance of the effect size were presented for each subgroup, and the difference in the mean Hedge's g values between the two groups was tested using the Z distribution. The subgroup analysis showed that RLS was significantly reduced in all studies, except for those without funding. Studies that received funding, used oil amounts of 10 mL or more, utilized commercial oil products, and had researchers or nurses as facilitators, had significantly greater reductions in RLS compared to their counterparts (Table 2).

To identify potential factors affecting the pooled overall effect of aromatherapy on RLS in hemodialysis patients, Univariate meta-regression was conducted. The results showed that there was a reduction in RLS when the study received funding and was conducted (Z = −5.03, *p* < .001). Additionally, when the study included more than 60 participants (Z = −2.17, *p* = .030), used lavender oil for aromatherapy intervention (Z = −2.45, *p* = .014), used 10 mL or more of oil, and used commercially available oils, there was a reduction in RLS (Z = −6.74, *p* < .001). Furthermore, when the intervention was conducted by researchers or nurses, there was a reduction in RLS (Z = 6.74, *p* < .001). There was a significant effect on reducing RLS with a quality evaluation score of less than 10 points (Z = 3.05, *p* = .002) in a study using Routine care for the Control group intervention (Z = 4.98, *p* < .001), with a mediation period of less than 4 weeks (Z = 3.01, *p* = .003), sessions lasting over 30 minutes each (Z = −3.94, *p* < .001), and delayed measurement time of the posttest (Z = 3.49, *p* < .001).

However, the application area and session of massage did not have a significant impact on reducing RLS (Table 3). Through the exclusion sensitivity test [22], when one study was excluded, Hedge's g was a large effect size ranging from −1.66 to −2.00. The 95% CI (−2.23 to −2.61, −1.09 to −1.41) did not include 0, all of which were statistically significant.

### Publication bias

**Figure 3.**
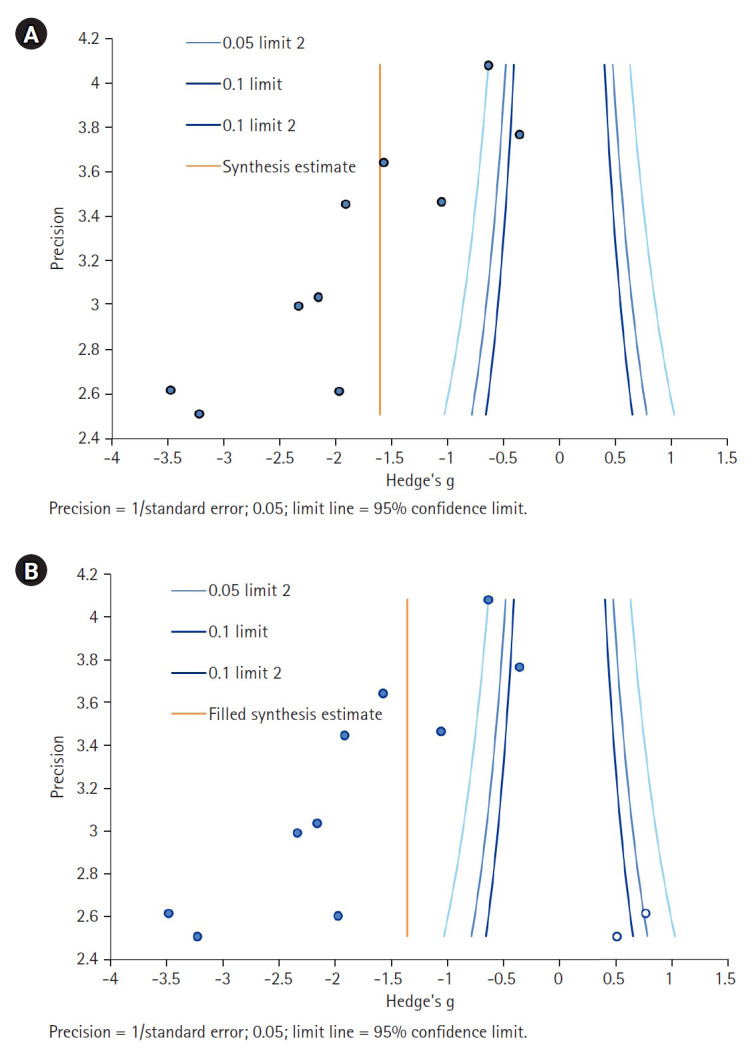
Funnel trim and fill plot of aromatherapy on restless leg syndrome among hemodialysis patients. (A) Funnel plot. (B) Trim and fill plot.

The results of the funnel plot and trim and fill plot analyses conducted to confirm the publication bias in the research showed that the individual effect sizes (blue circles) of the 10 studies included in this research were skewed to the left, indicating a somewhat asymmetric appearance, suggesting the presence of publication bias (Figure 3A). To address publication bias, the trim and fill plot indicated that two studies (white circles) should be added to the right side (Figure 3B). The trim and fill method [23] identified that two additional papers should be included in the study, resulting in a corrected effect size of 12 studies was −1.35 (95% CI: −1.53 to −1.17). Although the effect size of the reduction in RLS was slightly smaller after the correction compared to before, it still demonstrated a substantial effect size and remained statistically significant. In conclusion, the publication bias reported in this study was found to be at a level that the results regarding the reduction in RLS can be accepted even after correcting for the bias.

## DISCUSSION

The pooled overall effect of aromatherapy on RLS in hemodialysis patients was Hedge's g = −1.84 (95% CI: −2.45 to −1.23), indicating a large effect. This is consistent with studies that have shown aromatherapy to be beneficial not only in pain relief, providing antidepressant effects, promoting wound healing and blood circulation, but also in alleviating RLS symptoms and reducing RLS severity [24,25].

Regarding the results of the meta-regression analysis, the following points can be discussed:

First, the effect on RLS was more significant in studies with over 60 participants compared to those with fewer participants. This can be interpreted as the larger sample size offering ample statistical power to identify the effect. This suggests that obtaining a sufficient number of participants and ensuring satisfactory statistical power is crucial in research.

Furthermore, the use of lavender oil was significantly more effective for RLS than the use of other oils. This is consistent with previous studies that found significant effects of lavender oil on RLS in hemodialysis patients [26], and that lavender oil had positive impacts on itching, fatigue, muscle cramps, RLS severity, sleep, and quality of life in hemodialysis patients [27]. Aromatherapy massage stimulates the amygdala and hippocampus within the brain's limbic system, resulting in enhancements to physical, emotional, and mental well-being [28]. Lavender essential oil, which contains components such as linalyl acetate and linalool, is known for its pain management properties [29].

While various essential oils are commonly used in aromatherapy, such as lavender, rosemary, eucalyptus, chamomile, marjoram, jasmine, mint, and geranium [30]. Lavender oil, extracted from the lavender (*Lavandula angustifolia*) plant, is generally known to have beneficial effects on burns, insomnia, pain, anxiety, stress, skin problems, behavioral difficulties, and antimicrobial properties [31]. The results of this study indicate that among the various oils with different characteristics, lavender oil was particularly effective in treating RLS in hemodialysis patients.

On the other hand, when using lavender oil of 10 ml or more, the effect on RLS was significantly higher compared to when not using it. Additionally, when using commercialized oil, the effect on RLS was significantly higher compared to when not using it. When preparing directly without using standardized products, for example, mixing lavender oil (10 drops, 1.5%) and olive oil (10 drops) and applying it [27], or applying a 5% oil mixture (lavender, tea tree, almond, jojoba oil) 1 to 3 times on the itchy area without pores [32], various types of oils, ratios, and concentrations can be adjusted. In contrast, commercialized oil can be considered as standardized oil made with the most appropriate ratio. In this study, the use of commercialized oil was more effective than other oils. This was because it consistently demonstrated its effectiveness when combined with individual combinations, and it can be seen that it is most generally well-standardized when combined with products. Therefore, the results suggest that standardized mixing can enhance effectiveness. It was also observed that utilizing oil to its full capacity can be beneficial.

In terms of the duration of use, the findings suggest that using the oils for 4 weeks or less, with each session lasting 30 minutes or more, and having a control group receiving routine care, resulted in statistically significantly greater effects on RLS. In the duration is less than four weeks, the positive impact implies that the overall operating period is not significant. In addition, the fact that the effect was good when used for more than 30 minutes per session indicates the importance of being exposed to sufficient time of aroma therapy at once. That is, it is applied for a period of less than 4 weeks, but one session implies that spending more than 30 minutes is sufficient time. Generally, the application site, time, number of times, and total period are applied in various ways [26], and among them, it is necessary to develop the most appropriate protocol for the RLS of hemodialysis patients. In particular, the longer the exposure time, duration, and intensity of aromatherapy, the greater the effect; however, a shorter duration also enhances the effect. This finding suggests the presence of a cycle or curve that influences the effectiveness of aromatherapy. Therefore, it underscores the necessity for developing scientific and precise application protocols even for complementary and alternative therapies.

The finding that the effect was better when the control group received routine care suggests that the difference in effect may be minimal when other interventions are combined with aromatherapy. In other words, if other intervention programs were implemented, the difference in effect may not have been clearly observed. The most common methods used for RLS in hemodialysis patients are foot massage therapy and foot reflexology. Additionally, the effect size can vary depending on the type of massage. There are various complementary and alternative therapies available [33]. Since aromatherapy was utilized among these, the effect may have been more clearly observed when the control group received only routine care compared to when other interventions were used as the comparison.

Studies that received funding and those with a quality assessment score of 10 or less showed better effects on RLS. Funding and quality assessment scores are related to rigorous SD, so the inconsistent results in this regard warrant further research. This study is meaningful in suggesting the potential effectiveness of aromatherapy as an intervention for RLS, a common side effect experienced by patients undergoing hemodialysis for kidney disease.

## CONCLUSION

This study aimed to provide fundamental data on complementary and alternative therapies by conducting a systematic review and meta-analysis to examine the effect of aromatherapy on reducing RLS in hemodialysis patients. The pooled overall effect of aromatherapy on RLS in hemodialysis patients was found to be significant. The study is significant as it established the foundation for creating an efficient protocol for aromatherapy by examining the specific application methods and their effects.

## Figures and Tables

**Table 1.** Descriptive Summary of Restless Legs Syndrome in Hemodialysis Patients

Study ID	Author (yr)	Publi cation country	Number of centers	IRB	Fund	Participants	Research design	Oil type	Aroma oil preparation	E group intervention	C group intervention	Facilitator	Duration (week)	Number of sessions	Time/session (min)	Measurement time of post-test	Outcome variables (scale)
1	Ghasemi et al. (2021) [A1]	Iran	1	Yes	Yes	105 female HD patients (E^a^: 35, Eb: 35, C: 35)	RCT	E^a^: Lavender oil,	E^a^: linalool (27.11%) and linalyl acetate (23.33%) at a ratio of 3:3:2:2 mL in 100 mL of coconut carrier oil, 10 drops of lavender essential oil, Eb:6 drops of almond oil	Application: feet	[Placebo] Relaxing techniques, reflexology	Therapist	8	24	30	Immediately	- RLS severity (RLS rating scale)
								E^b^: Almond oil		-relaxing techniques, reflexology, aroma massage							
2	Amrollahi et al. (2022) [A2]	Iran	1	No	Yes	41 HD patients (E: 20, C: 21)	RCT	E: Lavender oil	Barij Essence Pharmaceutical Company.	Application: feet	Routine care	Nurses	4	12	30	Delayed	- RLS severity (RLS rating scale)
						- Female 18, Male 23			10 mL of lavender oil	-effleurage massage							
3	Ajorpaz et al. (2019) [A3]	Iran	2	Yes	Yes	90 HD patients (Ea: 29, Eb: 31, C: 30)	RCT	E^a^: Lavender oil,	E^a^: Barij Essence Pharmaceutical Company. 10~15 mL of 1.5% lavender oil, Eb: 10~15 mL of 2% glycerin	Application: legs	Routine care	Nurses	4	12	45	Delayed	- RLS severity (RLS rating scale)
						- Female 46, Male 44		E^b^: Glycerin		-effleurage massage							
4	Nasiri et al. (2019) [A4]	Iran	1	Yes	Yes	60 HD patients (E: 27, C: 28)	RCT	E: Olive oil	Loyeh Ind. 10 mL of olive oil	Application: lower legs and feet	[Placebo] liquid paraffin	Nurses	3	6	10	Delayed	- RLS severity (International RLS study group rating scale)
						- Female 27, Male 28				-massage							
5	Hashemi et al. (2015) [A5]	Iran	2	Yes	Yes	59 HD patients (E: 29, C: 30)	RCT	E: Lavender oil	Barij Essence Pharmaceutical Company. 10 to 15 mL with 1.5% lavender oil	Application: legs	Routine care	Nurses	3	6	10	Delayed	- RLS severity (RLS rating scale)
						- Female 31, Male 28				-effleurage massage							
6	Döner & Taşcı (2022) [A6]	Turkey	7	Yes	No	58 HD Patients (E: 31, C: 27)	RCT	E: Lavender oil	A 5 mL mixture containing 5% lavender oil was prepared by mixing with 100 mL of sesame oil	Application: lower legs	[Placebo] baby oil	Therapist	4	12	20	Immediately	- RLS severity (RLS rating scale)
						- Female 29, Male 29				-effleurage massage							- QoL of HD patients (KDQOL-36)
7	Oshvandi et al. (2021) [A7]	Iran	1	Yes	Yes	105 HD Patients (E^a^: 35, E^b^; 35, C: 35)	RCT	E^a^: Lavender oil,	E^a^: Barij Essence Co. 10–15 cc of 1.5% lavender oil, Eb: 10–15 cc of 1.5% sweet orange oil	Application: feet	Routine care	Researcher	3	9	30	Immediately	- RLS severity (RLS rating scale), sleep quality (PSQI)
								E^b^: Sweet orange oil		-effleurage massage							

Superscripts a and b were divided by intervention. IRB = Institutional review board; HD = Hemodialysis; E = Experimental group; C = Control group; RCT = Randomized controlled trials; RLS = Restless legs syndrome; QoL = Quality of life; KDQOL-36 = Kidney disease quality of life short form-36; PSQI = Pittsburg sleep quality index.

**Table 2.** Subgroup Analysis Regarding Restless Legs Syndrome in Hemodialysis Patients by Study Characteristics

Characteristics	Subgroup	K	Study ID	N	Overall ES	95% CI		Z (*p*)	Z (*p*)^†^
						Lower limit	Upper limit		
Funding	No	1	6	58	−0.36	−0.88	0.16	−1.35 (.177)	2.89 (.004)
	Yes	9	1^a^, 1^b^, 2, 3^a^, 3^b^, 4, 5, 7^a^, 7^b^	555	−2.00	−2.60	−1.41	−6.61 (< .001)	
Number of participants	< 60	3	2,5,6	158	−1.47	−2.69	−0.25	−2.37 (.018)	0.52 (.604)
	≥ 60	7	1^a^, 1^b^, 3^a^, 3^b^, 4, 7^a^, 7^b^	455	−2.00	−2.74	−1.25	−5.24 (< .001)	
Application area of the aroma massage	Feet	5	1^a^, 1^b^, 2, 7^a^, 7^b^	321	−1.88	−2.75	−1.02	−4.26 (< .001)	−0.09 (.931)
	Legs	5	3^a^, 3^b^, 4, 5, 6	292	−1.80	−2.78	−0.82	−3.60 (< .001)	
Oil type	Lavender	6	1^a^, 2, 3^a^, 5, 6, 7^a^	357	−2.10	−3.04	−1.16	−4.38 (< .001)	−0.73 (.462)
	Others	4	1^b^, 3^b^, 4, 7^b^	256	−1.46	−2.23	−0.70	−3.75 (< .001)	
Oil amount	< 10	3	1^a^, 1^b^, 6	198	−0.85	−1.55	−0.15	−2.38 (.017)	2.15 (.032)
	≥ 10	7	2, 3^a^, 3^b^, 4, 5, 7^a^, 7^b^	415	−2.27	−2.88	−1.67	−7.36 (< .001)	
Oil products	Hand-made	3	1^a^, 1^b^, 6	198	−0.85	−1.55	−0.15	−2.38 (.017)	2.15 (.032)
	Commercial	7	2, 3^a^, 3^b^, 4, 5, 7^a^, 7^b^	415	−2.27	−2.88	−1.67	−7.36 (< .001)	
Control group intervention	Routine care	4	2, 5, 7^a^, 7^b^	240	−2.36	−3.03	−1.68	−6.81 (< .001)	−1.16 (.246)
	Placebo	6	1^a^, 1^b^, 3^a^, 3^b^, 4, 6	373	−1.50	−2.27	−0.72	−3.78 (< .001)	
Facilitator	Researchers or nurses	7	2, 3^a^, 3^b^, 4, 5, 7^a^, 7^b^	415	−2.27	−2.88	−1.67	−7.36 (< .001)	−2.15 (.032)
	Aromatherapist	3	1^a^, 1^b^, 6	198	−0.85	−1.55	−0.15	−2.38 (.017)	
Duration (week)	< 4	4	4, 5, 7^a^, 7^b^	254	−2.12	−3.04	−1.20	−4.52 (< .001)	−0.53 (.593)
	≥ 4	6	1^a^, 1^b^, 2, 3^a^, 3^b^, 6	359	−1.65	−2.47	−0.83	−3.92 (< .001)	
Number of sessions	< 9	2	4, 5	114	−1.59	−2.67	−0.51	−2.88 (.004)	0.33 (.738)
	≥ 9	8	1^a^, 1^b^, 2, 3^a^, 3^b^, 6, 7^a^, 7^b^	499	−1.90	−2.65	−1.15	−4.97 (< .001)	
Time/session (min)	< 30	3	4, 5, 6	172	−1.17	−2.17	−0.18	−2.31 (.021)	1.08 (.280)
	≥ 30	7	1^a^, 1^b^, 2, 3^a^, 3^b^, 7^a^, 7^b^	441	−2.13	−2.86	−1.39	−5.67 (< .001)	
Measurement time of post-test	Immediately	5	1^a^, 1^b^, 6, 7^a^, 7^b^	338	−1.56	−2.51	−0.61	−3.22 (.001)	0.66 (.506)
	Delayed	5	2, 3^a^, 3^b^, 4, 5	275	−2.12	−2.82	−1.42	−5.97 (< .001)	
Quality score	< 10	5	3^a^, 3^b^, 6, 7^a^, 7^b^	318	−2.24	−3.38	−1.10	−3.85 (< .001)	−0.91 (.361)
	≥ 10	5	1^a^, 1^b^, 2, 4, 5	295	−1.44	−2.01	−0.87	−4.97 (< .001)	

The superscripts a and b indicate a division by the intervention. K = Number of analysis set; N = Number of participants; ES = Effect size; CI = Confidence interval; Quasi-E = Quasi-experimental study; RCT = Randomized controlled trial.

† Test of the difference between the average Hedge's g values of two groups.

**Table 3.** Meta-regression Analysis of Restless Legs Syndrome in Hemodialysis Patients

Coefficients (Ref.)	Estimate	SE	95% CI		*Z*	*p*
			Lower limit	Upper limit		
Fund ( No)	−1.43	0.29	−1.99	−0.87	−5.03	< .001
Number of participants (< 60)	−0.47	0.22	−0.89	−0.04	−2.17	.030
Application area of the aroma massage (Feet)	0.09	0.19	−0.29	0.47	0.46	.642
Oil type (Others)	−0.48	0.19	−0.86	−0.09	−2.45	.014
Oil amount ( < 10 mL)	−1.33	0.20	−1.71	−0.94	−6.74	< .001
Oil products (Hand-made)	−1.33	0.20	−1.71	−0.94	−6.74	< .001
Control group intervention (Routine care)	1.03	0.21	0.62	1.43	4.98	< .001
Facilitator (Researchers or nurses)	1.33	0.20	0.94	1.71	6.74	< .001
Duration (< 4 weeks)	0.60	0.20	0.21	0.99	3.01	.003
Number of sessions (< 9 sessions)	−0.09	0.24	−0.56	0.39	−0.37	.715
Time/session (< 30 minutes)	−0.81	0.21	−1.21	−0.41	−3.94	< .001
Measurement time of posttest (Delayed)	0.69	0.2	0.3	1.08	3.49	< .001
Quality score (< 10)	0.59	0.19	0.21	0.98	3.05	.002

Ref. = Reference; SE = Standard error; CI = Confidence interval.

## Data Availability

The data that support the findings of this study are available from the corresponding author upon reasonable request.

## Appendix 1. Search Strategy according to the PICO-SD Framework

**Table:**

PICO	Key terms	MeSH	PubMed Entry terms	Emtree (Embase)	Text words
P (patient, population, participants, problems)	Hemodialysis	"Renal Dialysis"[MeSH]	Dialysis, Extracorporeal	Hemodialysis/	Dialysis
			Dialysis, Renal		Hemodialysis
			Extracorporeal Dialysis		Haemodialysis
			Hemodialysis		Blood dialysis
					Blood purification
		"Hemodiafiltration"[MeSH]	Acetate-Free Biofiltration	Hemodiafiltration/	Hemodiafiltration
			Biofiltration, Acetate-Free		Haemodiafiltration
					Acetate-free biofiltration
		"Hemoperfusion"[MeSH]	Hemosorption	hemoperfusion/	Hemoperfusion
					Haemoperfusion
					Hemosorption
					Haemosorption
		"Hemofiltration"[MeSH]	Arteriovenous Hemofiltration	Hemofiltration/	Hemofiltration
			Venovenous Hemofiltration		Haemofiltration
					Blood filtration
		"Renal Replacement Therapy"[MeSH]	Kidney Replacement Therapy	Renal replacement therapy/	Renal replacement
			Replacement Therapy, Kidney		Kidney replacement
			Replacement Therapy, Renal		
			Therapy, Kidney Replacement		
			Therapy, Renal Replacement		
I (intervention or exposure or index test)	Aromatherapy	"Aromatherapy"[MeSH]	Aroma Therapy	Aromatherapy/	Aromatherap*
			Therapy, Aroma		Aroma therap*
					("oil" OR "aroma*") AND ("massage*" OR "topical*" OR "inhal*")
		"Oils, Volatile"[MeSH]	Essential Oil	Essential oil/	Essential oil*
			Oil, Essential		Volatile oil*
			Oil, Volatile		Aromatic oil*
			Oils, Essential		
			Volatile Oil		
				Fragrance/	Fragran*
					Scent*
		"Plant Extracts"[MeSH]	Herbal Medicines	Plant extract/	("plant*" OR "herb*" OR "lavend*" OR "rosemar*" OR "orange" OR "citrus") AND ("massage*" OR "topical*" OR "inhal*")
			Plant Extract		
		"Plants, Medicinal"[MeSH]	Healing Plants	Medicinal plant/	
			Herbs, Medicinal		
			Medicinal Herbs		
			Medicinal Plants		
			Pharmaceutical Plants		
Study design restrictions	RCT, Quasi-experimental				
	English, Korean / Humans (Adult: 19+ years), (Young Adult 19-24 years) Male, Female / 1900.01.01 - 2024.6.30				

PICO-SD = Population, intervention, comparison, outcome, study design; RCT = Randomized controlled trial.

## Appendix 2. List of Studies Included in the Systematic Review and Meta-Analysis

A1. Ghasemi M, Rejeh N, Bahrami T, Heravi-Karimooi M, Tadrisi SD, Vaismoradi. Aromatherapy massage vs. foot reflexology on the severity of restless legs syndrome in female patients undergoing hemodialysis. Geriatrics. 2021;6(4):99. https://doi.org/10.3390/geriatrics6040099

A2. Amrollahi A, Rafiei A, Bahri A, Nasiriani K. Effects of aromatherapy massage on the severity of restless legs syndrome in hemodialysis patients: a randomized clinical trial. Therapeutic Apheresis and Dialysis. 2022;26(6):1131-1136. https://doi.org/10.1111/1744-9987.13802

A3. Ajorpaz NM, Rahemi Z, Aghajani M, Hashemi SH. Effects of glycerin oil and lavender oil massages on hemodialysis patients’ restless legs syndrome. Journal of Bodywork and Movement Therapies. 2019;24(1):88-92. http://doi.org/10.1016/j.jbmt.2019.06.012

A4. Nasiri M, Abbasi M, Khosroabadi ZY, Saghafi H, Hamzeei F, Amiri MH, et al. Short-term effects of massage with olive oil on the severity of uremic restless legs syndrome: a double-blind placebo-controlled trial. Complementary Therapies in Medicine. 2019;44:261-268. https://doi.org/10.1016/j.ctim.2019.05.009

A5. Hashemi SH, Hajbagheri A, Aghajani M. The effect of massage with lavender oil on restless leg syndrome in hemodialysis patients: a randomized controlled trial. Nursing and Midwifery Studies. 2015;4(4):e29617. https://doi.org/10.17795/nmsjournal29617

A6. Döner A, Taşcı S. Effect of massage therapy with lavender oil on severity of restless legs syndrome and quality of life in hemodialysis patients. Journal of Nursing Scholarship. 2022;54(3):304-314. https://doi.org/10.1111/jnu.12738

A7. Oshvandi K, Mirzajani Letomi F, Soltanian AR, Shamsizadeh M. The effects of foot massage on hemodialysis patients' sleep quality and restless leg syndrome: a comparison of lavender and sweet orange essential oil topical application. Journal of Complementary and Integrative Medicine. 2021;18(4):843-850. https://doi.org/10.1515/jcim-2020-0121

## Appendix 3. Quality Assessment of the Included Studies

**Table:**

Study ID	Author (yr)	JBI of critical appraisal tools checklist for RCT													Total score (M ± SD)
		1	2	3	4	5	6	7	8	9	10	11	12	13	
1	Ghasemi et al. (2021)	1	1	1	0	0	1	1	1	1	1	1	1	1	11
2	Amrollahi et al. (2022)	1	0	1	0	0	1	1	1	1	1	1	1	1	10
3	Ajorpaz et al. (2019)	1	0	1	0	0	1	0	1	1	1	1	1	1	9
4	Nasiri et al. (2019)	1	0	1	1	1	1	1	1	1	1	1	1	1	12
5	Hashemi et al. (2015)	1	0	1	0	0	1	1	1	1	1	1	1	1	10
6	Döner & Taşcı (2022)	1	0	1	0	0	1	0	1	1	1	1	1	1	9
7	Oshvandi et al. (2021)	1	0	1	0	0	1	0	1	1	1	1	1	1	9
Total		7	1	7	1	1	7	4	7	7	7	7	7	7	10.00 ± 1.15

JBI = Joanna Briggs Institute; RCT = Randomized controlled trials; M = Mean; SD = Standard deviation.
